# Giant pilomatrixoma in the infraclavicular region following an insect bite

**DOI:** 10.1093/jscr/rjad182

**Published:** 2023-04-12

**Authors:** Juan P Cóbar, Sawsane Ghaddar, Markus G Feucht, Javier Ardebol

**Affiliations:** Department of Medical Research, Universidad Francisco Marroquín, Guatemala City, Guatemala; Department of Medical Research, Lebanese University, Beirut, Lebanon; Department of Medical Research, Universidad Francisco Marroquín, Guatemala City, Guatemala; Department of Medical Research, Universidad Francisco Marroquín, Guatemala City, Guatemala

## Abstract

Pilomatrixoma is a benign skin tumor typically presenting as a hard, slow-growing mass arising from hair follicle matrix cells. While most encountered in children, giant pilomatrixoma seldomly presents in adults. In the present case, a large subcutaneous, nonpainful and slow-growing mass was discovered in the infraclavicular region of a 52-year-old male. Biopsy confirmed the diagnosis of giant pilomatrixoma. Despite its benign nature, tumor size and location can result in significant morbidity and cosmetic deformity. This case highlights the importance of considering pilomatrixomas in patients with a slow-growing mass, especially after an inciting event, such as an insect bite. Timely diagnosis and proper management can result in successful tumor removal with minimal cosmetic compromise.

## INTRODUCTION

Pilomatrixomas are benign skin tumors originating from hair follicles, with minimal malignant potential in sporadic cases [1]. Some cases are linked to genetic syndromes like Gardner syndrome, myotonic dystrophy and Rubinstein–Taybi syndrome. Although the exact etiology is unknown, somatic mutations in the CTNNB1 gene have been reported in the most isolated pilomatrixomas [1]. In addition, factors such as trauma, surgery and vaccines have been associated in some cases [2].

Typically, pilomatrixomas present as an asymptomatic, firm, solitary, well-circumscribed tumor localized to the dermis or subcutaneous tissue, which usually arise in the head and neck regions. Tumors are described as giant when the tumor diameter exceeds 5 cm in its widest dimension [3–5]. Incisional biopsy remains the gold standard for diagnosis followed by histopathological analysis [4, 6, 7]. Once the diagnosis is established, surgical excision is most reliable with low recurrence rates [8]. Wide excision margins (i.e. 1–2 cm margins) are recommended in aggressive cases to reduce the risk of relapse [9].

The following case presents a giant pilomatrixoma arising in the infraclavicular region, after an insect bite, in an adult patient with successful surgical excision.

## CASE PRESENTATION

**Figure 1:**
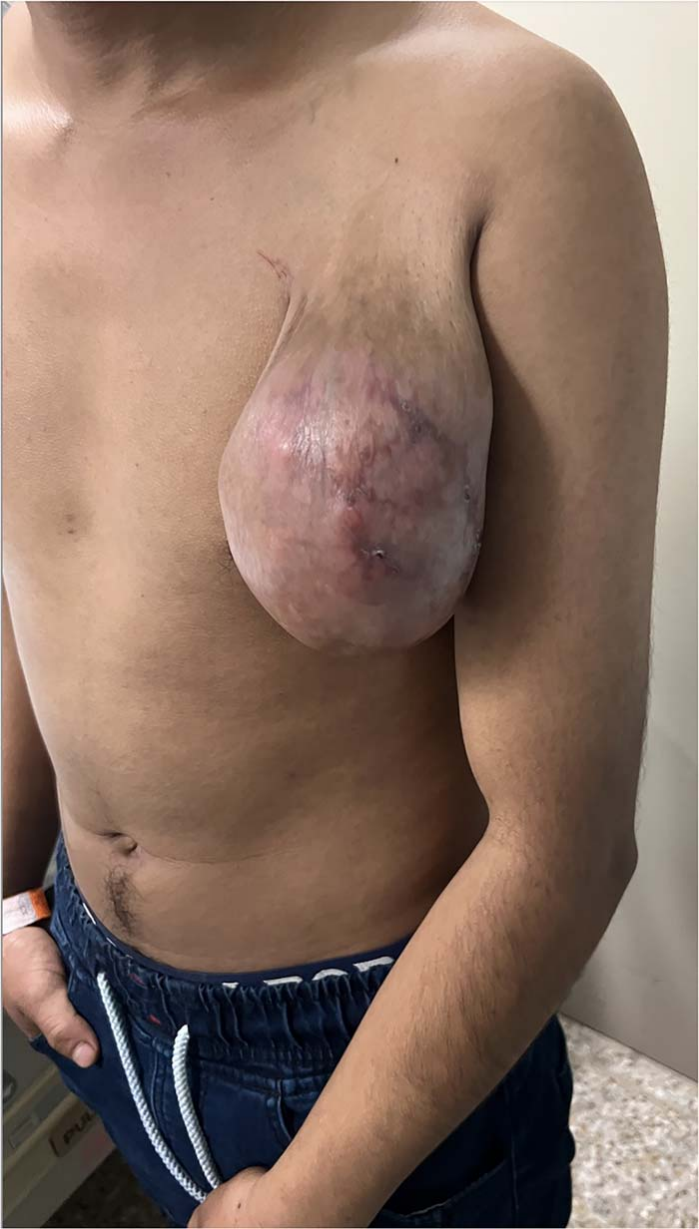
Left infraclavicular mass with small ulcerative lesions extending to the mid-arm level.

A 52-year-old male presented for a mass in the left infraclavicular region which had been gradually increasing in size for the past two years. The patient reports that a few weeks prior to noticing the mass, a mosquito, described as having legs with white stripes, bit him in the same region. Upon physical exam, a large mass was palpated in the infraclavicular area. The mass was nontender, mobile and firm. The overlying skin was mildly erythematous with small, partially healed ulcers (Fig. 1). There were no bruits or pulsations. Treatment options were discussed with the patient who elected to proceed with surgery based on size and cosmetic deformity.

**Figure 2:**
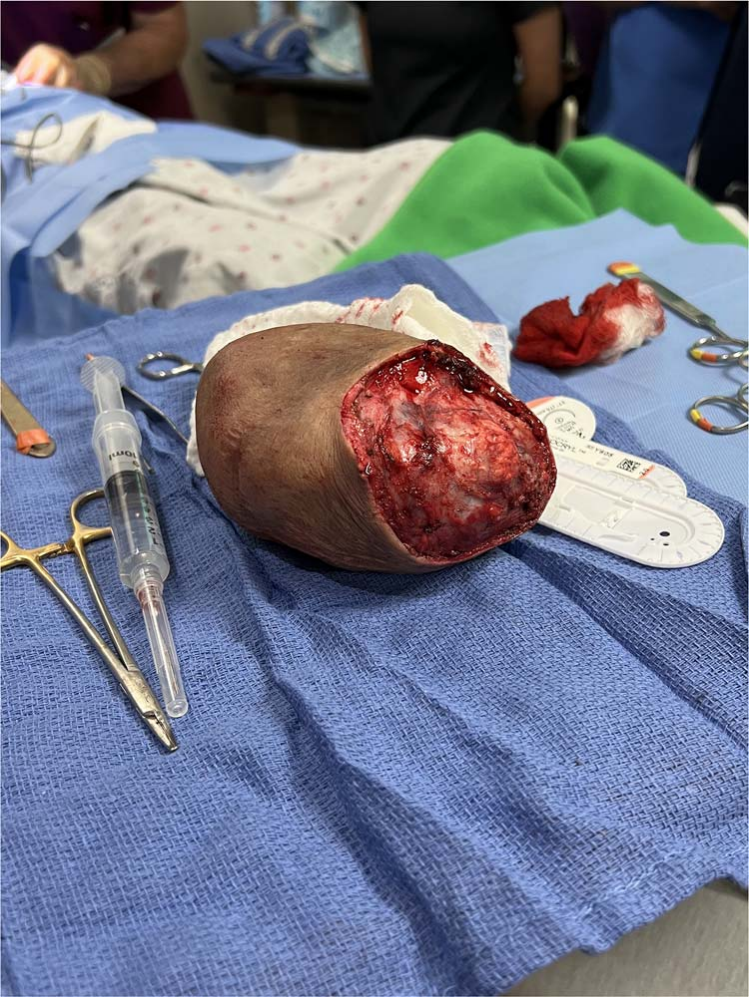
Resected left infraclavicular mass with 2 cm wide margins.

An en bloc resection of the mass was undertaken as follows. The patient was placed under general anesthesia, and the incision site was prepped with a local anesthetic. An elliptical incision was performed, followed by subcutaneous dissection. There was no infiltration past the subcutaneous layer as there was no need for fascial or muscular dissection. The mass was resected with 2 cm-wide margins and sent to pathology (Fig. 2). The incision edges were approximated using a two-layer closure. There were no complications in the early postoperative period.

**Figure 3:**
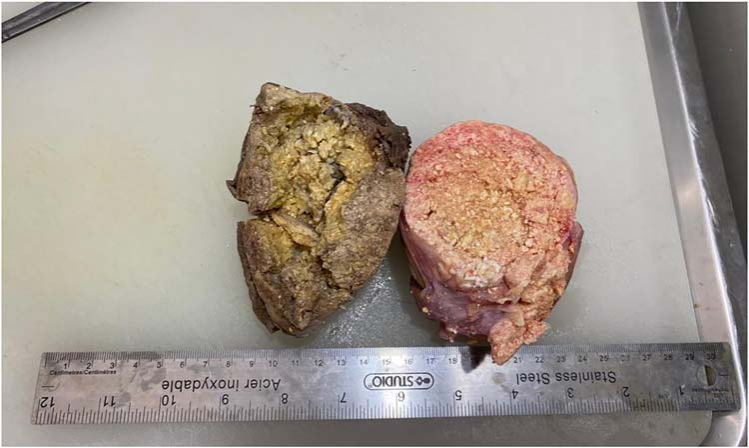
Incised mass revealing yellowish areas with a friable calcified appearance and solid uncalcified whitish areas consistent with foreign body reaction.

**Figure 4:**
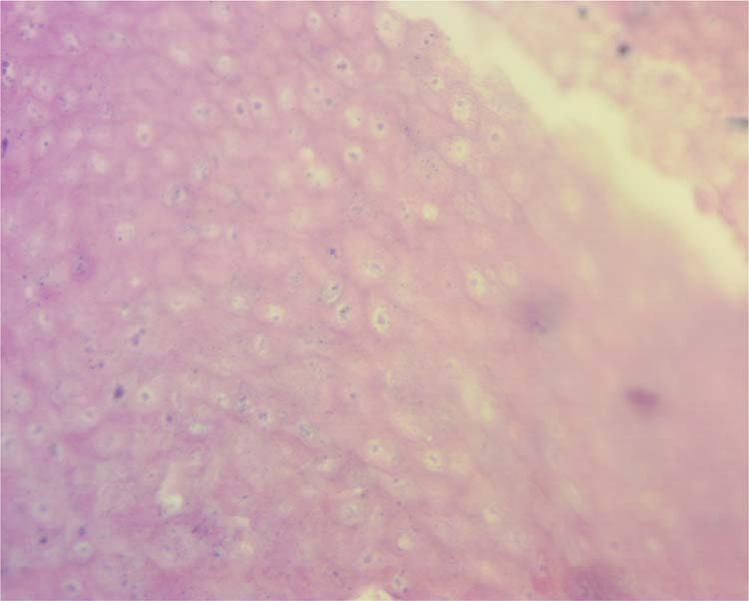
Tumor microscopy revealing basophilic cells that mimic the stratus basalis interspersed with characteristic ghost cells.

Macroscopically, the mass measured 20.1 * 13 * 5.8 cm. When incised, it showed a combination of yellowish areas with a friable calcified appearance and solid uncalcified whitish areas (Fig. 3). Histologically, sections were characterized by abundant ghost cells mixed with basophilic cells that imitate the cells of the basal layer of the hair follicle in the dermis (Fig. 4). There were extensive areas of calcification, necrosis and foreign body reaction, but no evidence of malignancy. These findings were most consistent with a giant pilomatrixoma.

## DISCUSSION

Our case matched the epidemiological profile of pilomatrixomas reported in the literature, which indicates a bimodal age distribution for this tumor, with the highest peak in the first and second decades and the second between 50 and 65 years of age [8]. According to Guinot-Moya *et al*., pilomatrixomas are slightly more common in males [8, 10], contrary to the findings of other studies [11, 12]. In addition, some studies have observed a familial pattern of presentation [13] However, in this case, no familial antecedent was reported.

The most significant case of a giant pilomatrixoma measured 34 cm × 21 cm × 17 cm [14]. It was resected from the posterior thorax of a healthy pediatric patient Another giant pilomatrixoma presented in a 52-year-old man on the posterior thorax and measured 24 cm × 21 cm × 9 cm [15]. In contrast, the mass in our case measured 20 cm × 13 cm and was located on the anterior thorax, precisely in the infraclavicular region. Despite these cases reporting masses in the thorax, pilomatrixomas usually develop on the head and neck regions [1, 14, 15].

Our patient has a history of a mosquito bite a few weeks prior to noticing the mass. According to the patient's description, the mosquito could have been Aedes spp, the vector responsible for transmitting dengue, Chikungunya, Zika and other arboviruses. Like most cases described in the literature, the tumor in our case consisted of a solitary lesion. On the other hand, pilomatrixomas may be associated with Gardner syndrome, myotonic dystrophy, Steinert syndrome, xeroderma pigmentosum, Turner's syndrome or sarcoidosis [2, 8, 13].

Pilomatrixomas lack specific clinical symptoms, which delay the diagnosis, primarily when it arises in unusual areas, especially following an inciting event regarded as innocuous. The accuracy of the clinical diagnosis of pilomatrixoma was 28.9% and 46%, according to Ciucā *et al*. and Pirouzmanesh *et al*., respectively [2, 12]. Imaging can help in the differential diagnosis by identifying calcifications, thus ruling out lymphatic and vascular tumors. However, biopsy remains the gold standard for diagnosis. The pilomatrixoma presents typically as a lobulated pattern, conformed of three cell populations arranged in a circular configuration with basaloid cells in the periphery, then transitional cells and enucleated shadow cells (ghost cells) in the center. Other characteristics include calcifications primarily found in the center of the mass and foreign body giant cells arising following a granulomatous response to ghost cells [3].

Surgical excision is the treatment of choice due to a low relapse rate of 0.3% and appreciable cosmetic results [12]. In addition, malignant transformation is extremely rare and has been reported in elderly subjects with a history of multiple excision attempts [6, 8].

Pilomatrixomas are benign, slow-growing tumors; however, they can grow to giant sizes leading to skin lesions and cosmetic deformity, as in our case. Therefore, a high index of suspicion is required to make an accurate diagnosis, especially when encountered in unusual locations and with an associated indolent history.
